# Propofol Modulates Early Memory Consolidation in Humans

**DOI:** 10.1523/ENEURO.0537-19.2020

**Published:** 2020-06-19

**Authors:** Daa Un Moon, Nazli Esfahani-Bayerl, Carsten Finke, Daniel J. Salchow, Mario Menk, Simon Bayerl, Richard Kempter, Christoph J. Ploner

**Affiliations:** 1 Department of Neurology, Charité – Universitätsmedizin Berlin, Berlin D-13353, Germany; 2 Department of Psychiatry and Psychotherapy, Charité – Universitätsmedizin Berlin, Berlin D-10117, Germany; 3 Berlin School of Mind and Brain, Humboldt-Universität zu Berlin, Berlin D-10117, Germany; 4 Department of Ophthalmology, Charité – Universitätsmedizin Berlin, Berlin D-13353, Germany; 5 Department of Anesthesiology, Charité – Universitätsmedizin Berlin, Berlin D-13353, Germany; 6 Department of Neurosurgery, Charité – Universitätsmedizin Berlin, Berlin D-10117, Germany; 7 Institute for Theoretical Biology, Department of Biology, Humboldt-Universität zu Berlin, Berlin D-10115, Germany; 8 Bernstein Center for Computational Neuroscience, Berlin D-10115, Germany; 9 Einstein Center for Neurosciences, Berlin D-10117, Germany

**Keywords:** general anesthesia, hippocampus, memory consolidation, propofol, synaptic consolidation, systems consolidation

## Abstract

Maintenance of memory across time is crucial for adaptive behavior. Current theories posit that the underlying consolidation process depends on stabilization of synapses and reorganization of interactions between hippocampus and neocortex. However, the temporal properties of hippocampal-neocortical network reconfiguration during consolidation are still a matter of debate. Translational research on this issue is challenged by the paucity of techniques to transiently interfere with memory in the healthy human brain. Here, we report a neuro-pharmacological approach with the GABA_A_ergic anesthetic propofol and a memory task sensitive to hippocampal dysfunction. Patients undergoing minor surgery learned word lists before injection of an anesthetic dose of propofol. Results show that administration of the drug shortly after learning (∼13 min) impairs recall after awakening but spares recognition. By contrast, later administration (∼105 min) has no effect. These findings suggest significant changes in memory networks very early after learning that are decisive for later recall. Propofol general anesthesia provides an experimental tool to modulate the first steps of hippocampus-mediated memory consolidation in humans.

## Significance Statement

Consolidation of memories depends both on mechanisms at the synaptic and the systems level. How and when these mechanisms interact is currently unclear. Here, we have used the anesthetic drug propofol to create a transient pharmacological “lesion” of the neural substrates of memory consolidation in humans undergoing minor surgery. Our results show that there is a brief time window after learning where hippocampus-dependent memories are susceptible to GABAergic modulation with propofol. Later recall appears to depend significantly on integrity of these first steps of memory formation. We infer that there is significant rearrangement of memory networks during the first hours after learning. Propofol general anesthesia provides an experimental approach to interfere with early memory consolidation in humans.

## Introduction

A defining feature of memory is the creation of cerebral representations that bridge temporal gaps between experience and behavior. It has been known since the 19th century that memory is not a static mental image of the past but rather a dynamic and re-constructive process that alters memory traces and involves distinct neural substrates as time proceeds (Ribot, 1882; Ebbinghaus, 1885). The mechanisms that stabilize memories have been termed memory consolidation (Müller and Pilzecker, 1900). A key clinical finding that has shaped the currently prevailing view on the neural substrates underlying consolidation is that some patients with lesions affecting the hippocampus show a temporally graded amnesia with relative sparing of remote memories, i.e., memories that were acquired months to years before hippocampal damage (Scoville and Milner, 1957). The “standard model” posits that consolidation involves a re-distribution of memories between hippocampus and neocortical networks with a decreasing role of the hippocampus with increasing memory delays (Alvarez and Squire, 1994; McClelland et al., 1995; Squire et al., 2004). However, the time scales addressed in patient studies of memory consolidation are not easy to reconcile with results from more recent imaging studies, showing that interactions between hippocampus and neocortex during the seconds and minutes that follow memory encoding are predictive of later recall (Tambini et al., 2010; Ben-Yakov and Dudai, 2011). To account for the wide range of memory delays, it has been suggested that consolidation should be seen as a family of processes on multiple time scales that transform, stabilize and update memory traces according to contextual demands (Dudai et al., 2015). Drawing largely from results from experimental studies in animals, it has been proposed that processes on a synaptic level at a time scale of up to some hours may provide iterative subroutines for consolidation on a systems level at much longer time scales (Dudai et al., 2015; Kukushkin and Carew, 2017; Asok et al., 2019).

It has proven difficult to provide complimentary experimental data for humans. There are virtually no studies that link clinical investigations in humans with hippocampal dysfunction and synaptic accounts of memory consolidation. An ideal patient model for the investigation of memory consolidation would consist of a transient brain lesion that acts selectively on a distinct phase of memory consolidation. However, most brain lesions are permanent and thus simultaneously affect encoding, consolidation and retrieval. Moreover, the hippocampus and surrounding structures are not accessible for current transcranial brain stimulation techniques. Modulation of long-term potentiation (LTP) by direct microstimulation of the human entorhinal cortex during memory tasks is a promising tool in this respect but limited to patients undergoing evaluation for epilepsy surgery (Titiz et al., 2017).

Here, we have taken a new neuro-pharmacological approach on human memory consolidation. We tested whether general anesthesia with the anesthetic propofol (2,6-diisopropylphenol) interferes with memory consolidation when applied shortly after learning and whether these effects are time dependent. Propofol is a short-acting anesthetic drug that is broadly used for sedation during invasive diagnostic and surgical procedures and for sedation in intensive care units (Sahinovic et al., 2018; Walsh, 2018). Propofol is both an agonist on GABA_A_ receptors and a partial antagonist on NMDA receptors. Studies in rat hippocampal slices suggest that these properties account for reduction of LTP and affect synaptic consolidation (Wei et al., 2002; Nagashima et al., 2005). Systemic administration of propofol immediately after learning of a location in a water maze has moreover been shown to affect consolidation of spatial memory in rats (Zhang et al., 2013).

Since ethical constraints limit experiments with anesthetic doses of propofol in healthy volunteers, we investigated patients undergoing minor ophthalmic surgery receiving propofol as a centrally acting drug during a short general anesthesia. Subjects performed a verbal learning and memory task that has previously proven to be sensitive to hippocampal dysfunction (Saury and Emanuelson, 2017). Verbal material was learned preoperatively at two different time points and tested postoperatively both for recall and recognition.

## Materials and Methods

### Participants

**Figure 1.**
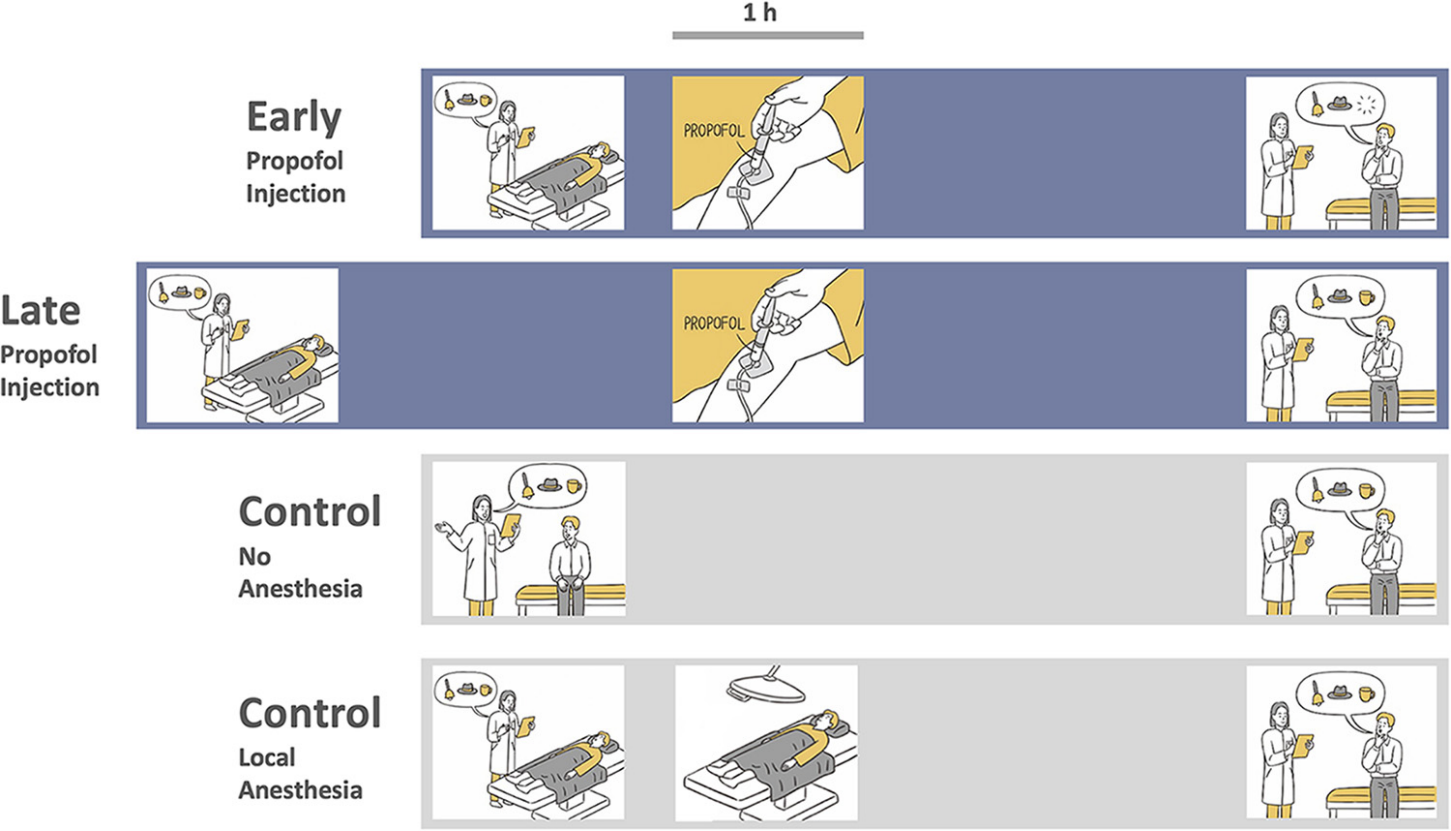
Task and experimental conditions. First row, Early injection condition. Second row, Late injection condition. Third row, Control condition. Fourth row, Local anesthesia condition. In all conditions, subjects learned a list of semantically unrelated and emotionally neutral words. In the early injection condition, subjects received general anesthesia with propofol ∼13 min following learning and were tested for recall and recognition about 3 h after learning. In the late injection condition, subjects received general anesthesia ∼105 min after learning and were tested ∼4.5 h after learning. In the control condition, subjects received no anesthesia and were tested 3 h after learning. In the local anesthesia condition, subjects received local anesthesia and were tested 3 h after learning.

We included subjects between 18 and 60 years of age without any history of neuropsychiatric disorders, hearing disorders or substance abuse. Four groups with a total of 96 subjects were tested (4 × 24 age-matched and sex-matched subjects; 49 females; Table 1). Two groups received general anesthesia with propofol for strabismus surgery at two different timepoints after learning (“early injection” and “late injection,” respectively; Fig. 1). In the early injection group, we aimed to act on early steps of memory consolidation immediately following learning. We thus kept the delay between end of learning and injection of propofol as short as possible. In the late injection group, it was aimed to act on a later phase of memory consolidation, i.e., clearly beyond the effects of propofol on maintenance of LTP in rat hippocampal slices (Wei et al., 2002) and longer than the expected duration of surgery/anesthesia in the early injection group (∼60 min). We thus aimed at a delay of ∼90 min between end of learning and injection of propofol. A third group consisted of healthy controls without any surgical procedure (control, no anesthesia; Fig. 1). A fourth group consisted of subjects undergoing local anesthesia for minor surgical procedures (control, local anesthesia; Fig. 1). This group was included to control for any presurgical arousal effects on our task (Pryor et al., 2010). All subjects spoke German fluently. Subjects undergoing surgery were recruited during preparatory visits in the outpatient departments of the Charité – Universitätsmedizin Berlin at least 3 d before surgery. Control subjects were recruited with advertisements via the intranet of the Charité – Universitätsmedizin Berlin. All procedures reported in this manuscript were approved by the ethics committee of the Charité – Universitätsmedizin Berlin. All subjects gave written informed consent before participation.

**Table 1:** Demographic and clinical data of the investigated patient groups

	Early injection	Late injection	Control, no anesthesia	Control, local anesthesia
*n*	24	24	24	24
Female/male	13/11	13/11	12/12	11/13
Age (years)	35.5 (27–45)	36.5 (31–47)	38.5 (25–46.25)	35 (29.25–46)
Years of education	13.75 (12.25–18)	14 (12–16)	16 (15–18)	15 (12–17)
Medical procedure	Strabismus surgery (*n* = 24)	Strabismus surgery (*n* = 24)	n.a.	Nevus excision (*n* = 13); muscle/nerve biopsy (*n* = 6);removal of ostheosynthetic material (*n* = 5)
Propofol bolus dose (mg)	200 (200–215)	200 (155–237.5)	n.a.	n.a.
Propofol maintenance dose (mg/kg/h)	6 (6–6)	6 (6–6.75)	n.a.	n.a.
Remifentanil dose (μ;g/kg/h)	0.2 (0.15–0.2)	0.2 (0.2–0.2)	n.a.	n.a.
Delay end of learning and Propofol (min)	13 (10–17)	105 (95.25–115)	n.a.	n.a.
Duration anesthesia (min)	58 (53–65)	56 (46.25–64.75)	n.a.	n.a.
Delay end of anesthesia and testing (min)	113.5 (106.5–128)	113 (108.5–116.75)	n.a.	n.a.
Delay end of learning and testing (min)	189.5 (175.75–205)	271 (261.25–289.5)	180 (180–180)	180 (150–180)

Values are medians and interquartile ranges; n.a., not applicable.

### Behavioral testing

Subjects were informed that they should perform a memory task before surgery and that they would receive a short additional testing after awakening from anesthesia. Subjects were not informed about the precise structure and purpose of the task and were not informed about the necessity to maintain to-be-remembered items across anesthesia. Subjects were tested with the Verbaler Lern- und Merkfähigkeitstest (VLMT; Helmstaedter and Durwen, 1990), a German version of the widely used Auditory Verbal Learning Test (AVLT; Lezak, 1983). None of the subjects was familiar with the task. In the learning phase of this test, the examiner read a list of 15 semantically unrelated and emotionally neutral words (e.g., “drum,” “coffee,” “river”) to the subject at a rate of one word every 2 s. After each presentation, the subject was requested to recall as many words as possible and to report all recalled words orally to the examiner. This list was presented five times to the subject and was each time recalled. After the fifth recall, a distractor list with 15 other words was presented and recalled. Afterwards, the original word list had to be recalled again. Learning took ∼15–20 min. Depending on the condition, subjects then received propofol general anesthesia, local anesthesia or were free to fill the delay until testing with intermediate activities. At testing, subjects were requested to recall the original word list and to report all recalled words orally to the examiner. Then, a recognition test was given. The examiner read a list which consisted of the 15 original words, the 15 words of the distractor list and 15 new words in pseudorandom order. For each word, the subject was requested to respond with “yes” or “no” whether the word had been part of the original word list.

### Procedure

In the early injection group (Table 1), subjects learned the word lists in a preparatory room adjacent to the operating theater, while being in a supine position. The time between end of learning and induction of anesthesia [median (Mdn) 13.0 min, interquartile range (IQR) 10–17] was filled with small talk with the examiner and explanatory remarks of the anesthesiologist. Then, subjects were preoxygenated with a face mask and received a bolus of propofol for induction of anesthesia (Mdn 200 mg, IQR 200–215) followed by a continuous infusion of propofol for maintenance of anesthesia (Mdn 6 mg/kg/h, IQR 6–6) and remifentanil for analgesia (Mdn 0.2 μ;g/kg/min, IQR 0.15–0.2). After loss of consciousness, the airway was managed with a laryngeal mask and subjects were mechanically ventilated. During anesthesia, subjects underwent surgery for ocular misalignment with recession, plication or resection of eye muscles according to established surgical standards (von Noorden and Campos, 2001). Anesthesia was continued for about 1 h (Mdn 58 min, IQR 53–65). After surgery, patients remained in a supine position until the end of testing. Apart from occasional communication with nurses and physicians, the postsurgical period was free of any specific activities. Postsurgical pain was treated with ibuprofen and paracetamol. Testing for delayed recall and recognition was conducted about 2 h after recovery from anesthesia (Mdn 113.5 min, IQR 106.5–128) and about 3 h after learning (Mdn 189.5 min, IQR 175.75–205).

In the late injection group (Table 1), subjects learned the word lists in a room on the ward, while being in a supine position. The time between end of learning and induction of anesthesia was filled with periods of rest, small talk with nurses and the examiner and explanatory remarks of the anesthesiologist (Mdn 105 min, IQR 95.25–115; *U *=* *0, *p* < 0.001 difference with early injection). Subjects maintained a supine position during the entire delay between learning and anesthesia. Subjects underwent the same surgical procedure and received a comparable dose of propofol (bolus: Mdn 200 mg, IQR 155–237.5; maintenance: Mdn 6.0 mg/kg/h, IQR 6–6.75; *U *=* *272, *p *=* *0.723 and *U *=* *235, *p *=* *0.108 difference with early injection group) and remifentanil as the early injection group (Mdn 0.2 μ;g/kg/min, IQR 0.2–0.2; *U *=* *218.5, *p *=* *0.093 difference with early injection group). Duration of anesthesia and postanesthesia recovery was like in the early injection group (Mdn 56 min, IQR 46.25–64.75; Mdn 113 min, IQR 108.5–116.75; *U *=* *251, *p *=* *0.445 and *U *=* *250, *p *=* *0.433 difference with late injection group). General postsurgical management was like in the early injection group.

In the control, local anesthesia group (Table 1), subjects learned the word lists in a preparatory room adjacent to the operating theater, while being in a supine position. The minutes between end of learning and local anesthesia (<10 min) were filled with small talk with the examiner and explanatory remarks of the surgeon. Depending on the surgical procedure, subjects then received local injections of lidocaine close to the region of surgery. Memory was tested after a 3-h delay (Mdn 180 min, IQR 150–180). General postsurgical management was like in the two propofol groups.

In the control, no anesthesia group (Table 1), subjects learned word lists in a seated position in a room on the ward. After learning, subjects were free to walk in the hospital, but were requested to return after ∼170 min. Testing was performed about 3 h after end of learning (Mdn 180 min, IQR 180–180).

### Experimental design and statistical analyses

All data obtained in this study are openly available at the Open Science Framework (osf) at https://osf.io/3x95n/. Data were analyzed by using IBM SPSS, version 25. Performance was described as percent correct responses in each subject. For initial learning, we analyzed the number of correctly recalled items from the original word list after presentation of the distractor list. For delayed recall, we analyzed the number of correctly recalled items from the original word list after the delay. For delayed recognition, we analyzed the number of correctly recognized items (hits) minus the number of erroneously recognized items (false alarms), thus yielding a “corrected recognition” value for each subject (Helmstaedter and Durwen, 1990). In order to analyze possible subtle impairments in source memory, we further separately analyzed false alarms to items from the distractor list and false alarms to new items. Group averages are given as Mdn and IQR. Since accuracy in behavioral tests is rarely normally distributed and since Kolmogorov–Smirnov testing showed that the assumption of a normal distribution had to be rejected (*p* < 0.05 for at least one subject group in learning, recall, and recognition conditions), non-parametric statistical testing was used for statistical analysis (Altman, 1991; Altman and Bland, 2009). Kruskal–Wallis ANOVA was used for analysis of group differences and two-tailed Mann–Whitney tests were used for *post hoc* comparisons between groups. Spearman rank order correlation was used for correlation analysis. Significance was accepted at a *p* < 0.05 level.

## Results

**Figure 2.**
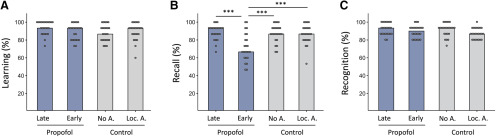
Results. ***A***, Free recall immediately after initial learning of target word list and after learning of a distractor word list. ***B***, Delayed free recall of target word list. ***C***, Delayed corrected recognition of target word list (hits minus false alarms). Bars show median percent correct responses in four experimental conditions. Purple, propofol injection conditions; gray, control conditions. No A., no anesthesia; Loc. A., local anesthesia; ***p ≤ 0.001 difference between conditions, two-tailed Mann–Whitney test. Note selective performance decrease for recall in the early injection condition.

After five repetitions of the original word list and presentation of the distractor list, all four groups showed similar retention of original word lists, with no significant differences between groups (χ^2^(3) = 6.204, *p *=* *0.102; Fig. 2). This suggests that presurgical arousal did not significantly affect initial learning of verbal stimuli. However, after the memory delay, significant group differences were found for recall of word lists (χ^2^(3) = 19.459, *p *<* *0.001; Fig. 2). Compared with control, no anesthesia and control, local anesthesia subjects, late injection patients showed unimpaired performance with no significant differences in recall of word lists (late injection, Mdn 93.3%, IQR 86.7–93.3; control, no anesthesia, Mdn 86.7%, IQR 81.7–93.3; control, local anesthesia, Mdn 86.7%, IQR 80.0–93.3; *U *=* *261.0, *p *=* *0.565 and *U *=* *241.5, *p *=* *0.332, respectively; Fig. 2). Any hangover effects of general anesthesia or the surgical procedure on recall are thus unlikely. By contrast, early injection patients showed a significant decrease in recall of word lists compared with control, no anesthesia subjects and late injection patients (early injection, Mdn 66.7%, IQR 60–85; *U *=* *123.0, *p *=* *0.001 difference with control, no anesthesia; *U *=* *107.5, *p *<* *0.001 difference with late injection; Fig. 2). Importantly, recall in early injection patients was also significantly different from the control, local anesthesia group (*U *=* *129, *p *=* *0.001 difference; Fig. 2). This result and the almost identical performance in both control conditions (*U *=* *274, *p *=* *0.767 difference; Fig. 2) suggest that presurgical arousal or some other direct reaction to the surgical procedure did not significantly affect initial consolidation of word lists.

Similar to previous observations of a differential susceptibility of delayed recall and recognition of word list learning to hippocampal dysfunction (Schoenberg et al., 2006; Finke et al., 2017), corrected recognition of word lists did not differ significantly between groups, although a statistical trend might have been present (χ^2^(3) = 7.363, *p *=* *0.061). Comparison of corrected recognition scores shows that this trend was mainly driven by a slightly lower performance of the control, local anesthesia group rather than by subtle performance deficits in the propofol groups (late injection, Mdn 93.3%, IQR 86.7–100.0; early injection, Mdn 90%, IQR 81.7–93.3; control, no anesthesia, Mdn 93.3%, IQR 86.7–93.3; control, local anesthesia, Mdn 86.7%, IQR 80–93; Fig. 2). Moreover, when hit rates and false alarms were analyzed separately, no significant differences were found between groups (hit rate, χ^2^(3) = 4.733, *p *=* *0.192; false alarms to items from the distractor list, χ^2^(3) = 1.626, *p *=* *0.653; false alarms to new items, χ^2^(3) = 2.926, *p *=* *0.403).

In order to analyze the selectivity of the recall–recognition dissociation in early injection patients, we next compared difference between recall and corrected recognition in all four groups (“Δ-R-R”). As expected, there was a significant difference of Δ-R-R between groups (early injection, Mdn 20%, IQR 6.67–33.33; late injection Mdn 0%, IQR −5.0–6.67; control, no anesthesia, Mdn 0%, IQR −5.0–13.33; control, local anesthesia Mdn 0%, IQR 0–0; χ^2^(3) = 25.111, *p *<* *0.001 difference between groups). *Post hoc* testing further showed that there was a significant difference of Δ-R-R between early injection and all other three groups but not between the other three groups (early injection vs all other groups, *U *≤* *146, *p* ≤ 0.003; all other comparisons, *U *≥* *221, *p* ≥ 0.157). This analysis shows that the difference between recall and corrected recognition is selective for the early injection group.

Because of the clinical setting, subjects both in the early injection and late injection condition showed some variability in time between end of learning and injection of propofol (ranges: early injection, 6–21 min; late injection, 72–140 min). To more precisely infer on a possible time window for propofol effects on word list consolidation, we tested whether recall performance showed a relationship with time to injection in both groups. However, we found no significant correlation between these variables when calculated separately for both groups (early injection, *r* = 0.286; *p* = 0.175; late injection, *r* = −0.007; *p* = 0.975), thus suggesting that susceptibility of word list consolidation to propofol general anesthesia ends at some time point between 21 and 72 min following learning.

## Discussion

The findings of our study show that propofol general anesthesia interferes with declarative memory in a task that is commonly used to assess integrity of the human hippocampus. The amnesic effect of propofol general anesthesia is critically time dependent and appears to be limited to a brief time window following learning. We infer that propofol general anesthesia modulates presumably hippocampus-dependent initial steps of memory consolidation.

Since its approval at the end of the eighties of the last century, propofol has become a dominant anesthetic agent for induction and maintenance of general anesthesia, ambulatory surgical procedures and sedation in intensive care patients (Sahinovic et al., 2018; Walsh, 2018). Propofol has a rapid onset and is quickly eliminated. With infusions of a duration of 1 h, the context-sensitive half-time of propofol is <10 min (Hughes et al., 1992; Sahinovic et al., 2018). Clinically, this accounts for rapid recovery times compared with other anesthetics (10–30 min). Apart from its clinical applications, propofol has increasingly been used as a recreational drug (Xiong et al., 2018). Since soybean oil is used as a solubilizer, propofol has a milk-like appearance and has thus been nicknamed the “milk of amnesia” (Walsh, 2018). Although this sobriquet implies some interference of the drug with memory processes, there are surprisingly few experimental investigations of propofol effects on the neural substrates underlying memory formation.

LTP and long-term depression (LTD) of synaptic transmission are thought to represent key mechanisms underlying transformation of labile representations of perceptual input into longer-lasting memories (Martin et al., 2000; Takeuchi et al., 2014). Recordings of EPSPs from the CA1 region of the rat hippocampus have shown that an injection of propofol transiently (<60 min) inhibits field EPSPs in CA1 and affects maintenance of LTP, if given after LTP induction (Wei et al., 2002). Additional experiments on rat hippocampal slices showed that propofol can also inhibit induction of LTP and that this effect can be blocked by agents that block GABA_A_ receptors, but not by agents that block NMDA receptors (Nagashima et al., 2005). GABA_A_ receptors are densely expressed in the hippocampus and the deep layers of the cortex where they are pivotal for learning and memory, with some isoforms being particularly important for memory formation (Engin et al., 2018). Pharmacological modulation of GABA_A_ receptors has moreover been shown to affect memory consolidation-related sharp wave-ripple complexes in hippocampal networks. For example, at clinical concentrations, the anesthetic thiopental affects the incidence, rhythmicity and synchrony of sharp waves and the quantity of ripple oscillations in the CA1 region of hippocampal slices (Papatheodoropoulos et al., 2007). These effects appear to be mediated by distinct subunits of GABA_A_ receptors. In particular, α5GABA_A_ receptors appear to reduce hippocampal excitability and may inhibit memory formation (Engin et al., 2018). Accordingly, stimulation of α5GABA_A_ receptors with therapeutic concentrations of diazepam has been shown to reduce the number, duration and power of ripple oscillations and to produces a partial temporal dissociation between ripples and sharp waves (Koniaris et al., 2011). Application of high concentrations of diazepam can also reduce the frequency of sharp waves (Viereckel et al., 2013). Computational modeling of the effects of various GABAergic drugs suggests that changes in power and duration of ripple oscillations reflects altered dynamics of interneuron networks in the CA1 region of the hippocampus (Donoso et al., 2018). Correspondingly, when propofol is systemically administered to rats immediately after learning of a location in a water maze, memory retention 24 h following learning is impaired in a dose-dependent way (Zhang et al., 2013).

While these findings suggest that propofol should act on consolidation of human memory too, a transfer of these results on clinical settings has not been successful so far. It has been controversial whether it is possible to induce deficits in preoperatively learned material by subsequent administration of anesthetic agents (Veselis, 2018). Early experiments showed that sedative doses of propofol, i.e., doses that leave subjects able to communicate and breathe spontaneously, may affect memory of visual and verbal material, when stimuli are learned and tested during a continuous infusion of the drug, with effects being largely independent of the level of sedation (Veselis et al., 1997). Subsequent experiments with event-related potential recordings (ERPs) from subjects performing a continuous picture recognition task during propofol infusion showed a selective drug effect on pictures that were tested after 27 s, but not after 6 s (Veselis et al., 2009). ERP amplitudes during recognition decreased in parallel. More recently, functional magnetic resonance imaging (fMRI) during encoding of emotional pictures and continuous propofol infusion showed suppression of hippocampal responses that correlated with the degree of memory impairment for the stimuli (Pryor et al., 2015). While these studies make a strong point for modulation of memory-related neural activity in the human hippocampus by sedative doses of propofol, their focus was on revealing the mechanisms and the prevention of surgery-induced posttraumatic stress disorder (Pryor et al., 2015). Thus, it is difficult to disentangle the relative contributions of encoding, consolidation and retrieval to the antegrade amnesia induced in these experiments.

A critical prerequisite for studies of the time course of consolidation with anesthetic agents like propofol is the induction of retrograde memory effects, i.e., effects on material that is learned before infusion of the drug and tested after discontinuation. So far, there has been no convincing evidence for anesthetic-induced retrograde amnesia (Veselis, 2018). A previous study on patients with depression however successfully used electroconvulsive therapy (ECT) in deep anesthesia as an intervention to study reconsolidation of emotionally negative stories learned one week before treatment (Kroes et al., 2014). Recall of these stories was impaired, when memory of the story was cued immediately before ECT and tested 24 h afterward. By applying the same behavioral paradigm to patients receiving sedation for endoscopy, a recent study showed that propofol at sedative doses may induce similar, albeit slightly weaker, effects on reconsolidation of emotional story contents (Galarza Vallejo et al., 2019).

Our results add significantly to previous work by showing that propofol general anesthesia can indeed exert retrograde amnesia for emotionally neutral declarative to-be-remembered items. Normal performance in the late injection condition of our study suggests that the amnesic effect of propofol general anesthesia may extend up to ∼30–60 min before injection. This new finding suggests that propofol general anesthesia acts on postencoding processes that are decisive for initial consolidation and later recall. Electrophysiological signatures of early memory formation have been found in direct recordings of ERPs from the hippocampus of patients undergoing evaluation for epilepsy surgery. ERPs recorded during learning of word lists separated subsequently recalled from unrecalled words (Fernández et al., 1999). Studies with fMRI have further shown that interactions between hippocampus and neocortex during the minutes that follow encoding of visual associative stimuli are predictive of later recall (Tambini et al., 2010). Similarly, activity in hippocampus and caudate nucleus following stimulus offset can predict memory of audiovisual episodes (Ben-Yakov and Dudai, 2011). Despite the heterogeneity of approaches, these and related studies therefore provide evidence for a pivotal role of the hippocampus for the very first steps of declarative memory consolidation.

It must be conceded that clinical propofol anesthesia is always administered in the context of invasive procedures, mostly in combination with intravenous opioid analgesia. Whether this might have contributed to the deficits in the early injection condition of our study remains elusive. A recent review concluded that opioid signaling is not required for, but can sometimes act to constrain, hippocampus‐dependent memory (Thomas, 2015). Likewise, it is possible that arousal before a surgical procedure may influence memory consolidation (Pryor et al., 2010; Chen et al., 2016). We deem this factor not to be decisive, at least for the task in our study, as surgery in local anesthesia did not produce a memory impairment. We are therefore confident that the effects on early consolidation observed here are mainly attributable to pharmacological actions of propofol.

One reason why previous pharmacological studies did not reveal the same retrograde effects observed here may be lower serum concentrations of propofol in experiments with sedative doses of propofol in cooperative and spontaneously breathing normal subjects. In a study on reconsolidation of emotional story contents, retrograde propofol effects on reactivated memory of stories before propofol sedation were observed when subjects were tested 24 h after anesthesia, but not when tested after a delay of up to 106 min (Galarza Vallejo et al., 2019). Compared with this study, the anesthetic doses applied to our patients are significantly higher. At least in animal experiments, propofol effects on LTP are critically dose dependent (Wei et al., 2002; Nagashima et al., 2005). fMRI studies on pain processing at different propofol concentrations have moreover shown that connectivity changes within cerebral large-scale networks are critically dose dependent (Lichtner et al., 2018). A further point may be a differential sensitivity of the mnemonic representations across tasks to GABA_A_ergic drugs and to altered neuronal activity in distinct brain regions. The task used here has proven to be a reliable marker of hippocampal integrity, particularly for its recall component (Saury and Emanuelson, 2017). Thus, although propofol general anesthesia is likely to act on a wide network of brain regions, the pattern of results is most consistent with modulation of hippocampal neural activity (Finke et al., 2017; Esfahani-Bayerl et al., 2019). Predominant effects on recall in our experiments and the abovementioned reconsolidation study (Galarza Vallejo et al., 2019) further show that application of propofol after memory encoding does not lead to an unselective impairment but rather tends to affect some memory domains more than others, presumably sparing less hippocampus-dependent routes of memory consolidation.

Which level of consolidation has been modulated in our experiment? The time window identified in our study is suggestive of propofol actions on synaptic memory consolidation (Dudai et al., 2015; Asok et al., 2019). Systems and synaptic levels of memory consolidation have traditionally been considered separately and with distinct experimental approaches. It is only recently that the interaction between these two levels has been discussed within a common conceptual framework (Dudai et al., 2015; Asok et al., 2019). Current models of synaptic consolidation propose mechanisms by which synaptic plasticity impacts on memory-guided behavior at various timescales, including the short delays addressed here (Ziegler et al., 2015). Complimentary data from humans have been scarce so far. While it is of course not possible to infer from our behavioral results on modulation of synaptic and/or systems levels of memory consolidation, combination of the neuropharmacological approach of our study with imaging techniques may provide a way to link synaptic and systems consolidation in humans.

### Conclusion

The results of our study show that propofol general anesthesia may create a transient pharmacological “lesion” of the neural substrates supporting early memory consolidation. The lack of effect beyond this time window further suggests rapid subsequent reconfiguration of hippocampus-dependent memory networks. While our approach is spatially not selective, it nevertheless circumvents restrictions of traditional patient-based approaches and makes the initial steps of memory consolidation accessible to experimental modulation, without affecting encoding or memory retrieval. Importantly, it allows for the study of memory consolidation in human subjects with brains that are unaltered by neuropsychiatric disorders or brain surgery. Combination of propofol general anesthesia with subsequent functional imaging of memory replay in the hippocampus may ultimately reveal how transient modulation of GABAergic neurotransmission affects mechanisms of memory consolidation in humans.

## Synthesis

Reviewing Editor: Bradley Postle, University of Wisconsin Decisions are customarily a result of the Reviewing Editor and the peer reviewers coming together and discussing their recommendations until a consensus is reached. When revisions are invited, a fact-based synthesis statement explaining their decision and outlining what is needed to prepare a revision will be listed below. The following reviewer(s) agreed to reveal their identity: Arielle Tambini, Bryan Strange. Note: If this manuscript was transferred from JNeurosci and a decision was made to accept the manuscript without peer review, a brief statement to this effect will instead be what is listed below.

Here is the full text of the two reviews:

R1

The manuscript by Moon and colleagues reports a novel approach for manipulating humans memory consolidation during an early post-encoding period. Anesthetic doses of propofol were given to patients in the context of surgical procedures, with two groups receiving propofol at different times after initial word learning took place. These two propofol groups were compared to a control group (no anesthesia or surgery) and a group that underwent a more minor procedure that only required a local anesthetic. The results show that recall of words learned was impaired in the group that received propofol and underwent surgery shortly after encoding (∼15 minutes) compared to all of the other groups. A measure of ‘recognition’ memory did not differ across groups (but see below on this). The results are interpreted with regard to disrupting synaptic plasticity as well as systems-level consolidation processes. I think the result is interesting and appears to be striking, which will contribute to the field. This is important since there are not many ways to manipulate post-encoding consolidation processes in humans. However, I think there are several issues with the manuscript that need to be addressed prior to publication.

1) The results show reduced memory for the early-propofol group measured via recall but not in the recognition measure. First, regarding the nature of the recognition test, please clarify how the test was conducted. It is indicated in the Methods that participants had to identify which 15 of 45 words were from the learning phase (15 new words, 15 distractors, and the learned words were presented). Yet the specific decision made for each stimulus is not described (e.g. Yes or No, if it was from the initial learning phase, an Old or New judgement, or a three-alternative forced choice between the learning phase, distractor phase, or New). The format of the test will indicate what measures can be extracted from the data, i.e. if the test is capable of measuring source memory (whether the old stimuli belonged to the learning or distractor lists) versus simple old/new item recognition. It is also not clear what measure is reported from this recognition test (it is not described in the Methods): is accuracy collapsed across all trials, or is the hit rate being reported? This needs to be clarified, and depending on how the test was conducted, hit and false alarm rates should be reported to fully understand whether memory differs between experimental groups. For example, it is possible that a more subtle difference in source memory may be present (ability to distinguish between words from the learning and distractor phases) but measuring accuracy across all trial types could obscure this kind of pattern.

Secondly, the authors interpret their results as showing that early propofol selectively influences recall and not recognition memory, which could point towards a greater influence of early propofol on memory that is more supported by hippocampal function (recall) versus item recognition which can be supported by extra-hippocampal structures. Although I understand why the authors want to draw this link to show some specificity in their findings, it is currently not supported by the data because (1) of the nature of the recognition memory test (see prior point) and (2) the results are not statistically compared between the recall measures and recognition measures to show that the difference in recall is significantly different than the lack of difference in recognition. In other words, a memory type by group interaction should be present to make the claim of selectivity.

2) The authors conclude that their approach of propofol administration may provide a novel method for linking synaptic consolidation and systems consolidation in humans. It is very much unclear and an open question (at least to me) how this approach would link these processes. While it is understood that propofol is a GABA-A agonist, and as described in the manuscript, has been shown to influence synaptic plasticity in hippocampal slices, it is also plausible that propofol may modulate active mechanisms that are thought to promote systems-level consolidation (i.e. hippocampal-cortical interactions and hippocampal ripple events and reactivation). It seems likely that all of these putative consolidation processes (both at the synaptic and systems level) would be influenced by propofol. It thus seems difficult to claim that this approach could somehow bridge these processes, unless the authors mean that it could broadly influence many possible mechanisms that may contribute to long-term retention. This could be desirable from a clinical or therapeutic perspective, but it seems difficult to be able to infer the specific mechanisms that contribute to memory impairment after propofol administration (especially without measuring any of these putative mechanisms).

Minor points:

3) The patient groups used in the study were given propofol in the context of undergoing surgical procedures. Yet, in the Methods, none of the surgical procedures are even briefly described. I understand that this is not the main manipulation of interest, yet it is still a part of the experimental procedure and so needs to be described at least briefly (with references) in the Methods. Were any drugs other than propofol administered that could act on the CNS? The Discussion also fails to mention that propofol was administered in the context of patients undergoing surgical procedures - this detail should not be omitted here.

4) Related to the prior point, several parts of the experimental timeline should be better described. Specifically on pg. 8 it is stated, that after learning “Depending on the condition, subjects then received propofol general anesthesia, local anesthesia, or were free to fill the delay until testing with intermediate activities.” Please describe these time periods in more details. In the groups that underwent surgery, what were participants doing in between learning and the time of when their procedure began? Did the participants in the control group (that did not undergo surgery) leave the lab in between learning and memory testing? Please describe their conditions as best as is possible (note that the nature of post-encoding activities can influence memory retention, see Dewar et al., 2014, Brokaw et al., 2016).

5) Since free recall was performed both at the end of learning and after the delay, I was surprised that the authors did not estimate a measure of memory retention (i.e. delayed over immediate memory), given the clear focus on consolidation. This would help to compare a normalized measure of memory across groups, given that there is considerable across-participant variance in memory which would only obscure across group differences due to experimental manipulations. This is not a major issue since there is are robust differences (in the early propofol group vs. other groups) during delayed testing, but, by controlling for initial memory, this may reveal other differences between groups as well which could be obscured by across-participant variance in initial learning. This measure also more closely matches the theoretical interest in consolidation.

6) Please include a description of the time course of propofol in terms of its influence of cognition - this is relevant since memory testing is administered at different times relative to its administration, but no details are provided. This is important for the claim that propofol is selectively influencing a consolidation phase (and not retrieval).

7) It would also be helpful if the authors could include any ratings of participant’s behavioral state before/after having undergone surgical treatments. Were there any differences in stress and arousal levels from learning to memory testing across the groups? For example, given that any surgical procedure likely acts as a stressor, it is surprising to me that there is no apparent difference in memory retention between the control group and the minor surgical procedure group (w/o propofol), or the group with delayed propofol administration, given that a large body of work indicates that stressors after encoding influence later memory. Any stress or mood ratings would help to clarify what kinds of differences in behavioral states were present during memory testing across the groups. Of course, given that there is a difference in recall between the early and late propofol groups in this study, I do not think that this a major issue (as presumably stress levels or other changes in mood should be similar across these groups) but it would be helpful to verify this if possible.

8) In the Methods, the duration of word presentation and ISI during the learning phase is not described - were the words presented at a rate of 1 per second (I believe this is typical for the AVLT)?

9) In the Discussion, the authors indicate that “Pharmacological modulation of GABA_A receptors” ... can “change” properties of sharp wave ripple events in the hippocampus. Please be more specific when describing the prior literature - this text could be more informative to the reader if the authors describe how different agents act on SWRs i.e. whether agonists increase or decrease the rate of ripples, etc. The reader is left wondering whether propofol or other agonists would disrupt ripples (i.e. by decreasing the incidence and/or frequency, or synchrony during ripples, etc.).

10) In the Discussion (same paragraph as the above point) the authors mention that systemic, post-encoding propofol administration in rodents disrupts retention of learned locations in the Morris water maze. It would be beneficial to mention this paper in the Introduction as it clearly helps to motivate the current study.

11) Figure 1 could be improved by including an approximate time scale of the events.

R2

Ploner and colleagues report interesting findings regarding the ability of the general anaesthetic propofol to block early, but not late, consolidation of auditory verbal memories. Instead of subjecting individuals to an anaesthetic they would otherwise not need, the authors performed their experiments in patients undergoing ophthalmic surgery. The “early” group received propofol ∼14 mins after encoding whereas a “late” group underwent anaesthesia after ∼100 mins. Control groups included one group undergoing a procedure with local anaesthesia, and a further group who had no anaesthesia. The primary result is that the early group showed reduced free recall relative to all other groups.

These are not easy experiments to coordinate in the clinical setting and I compliment the authors on their approach.

1. During encoding, a distractor list is presented after the 5th recall of the original list, and recall tested. The original list is then recalled again (Trial 6). However, in the results section, we are only told that after five repetitions, all four groups showed similar retention of word lists, with no significant differences between groups. As retroactive interference may differ between individuals, it is important to state the recall scores for Trial 6, and ensure that these do not differ between early and late propofol groups.

2. The recognition score are at ceiling (a large proportion of subjects score 100%). However, the authors have not provided hit rates that are corrected for false alarms (responding ‘old’ to new items). Indeed, we are not told what the exact instructions were for the recognition task. Were these push-button or oral responses? Were these spoken or written words? Was the judgement to be made ‘original old’ vs. ‘not original old’? Again, we would want to see the false alarm rate for the distractor list.

3. Please indicate the (absence of) significant differences for key parameters such as Propofol bolus/maintenance doses, Remifentanil dose, duration of anaesthesia, etc., either in the main text or in Table 1.

4. The anesthetic doses applied in the current study are significantly higher than previous studies at sedative doses. It would be worthwhile adding a section in the Methods to explain these details here. How was the airway managed?

Minor points

-Introduction “Since ethical constraints do not permit experiments with anesthetic doses of propofol in healthy volunteers”. Is this the case in all countries?

-Strictly speaking, the experimental design in terms of times of delay until propofol/testing etc. should be provided in the methods section.

-In the results, Mann Whitney U should be reported with median values (as per APA guidelines)

-I did not understand the last line of the results section: “...suggesting that susceptibility of word list consolidation to propofol general anesthesia ends at some time point between 14 and 104 minutes following learning”. If the interval between study and early injection is as long as 20 mins, and delay does not correlate with consolidation effects, shouldn’t this be 21 instead of 14?

-In the Discussion, “One of the prerequisites for memory consolidation is a persistent change in synaptic strength following learning.” Not everyone agrees with this.

-Figure 1 would be improved by including the (approximate) delays between conditions for each group.
